# Poly[(μ_2_-4,4′-bipyridine)bis­(μ_4_-5-*tert*-butyl­isophthalato)bis­(μ_3_-5-*tert*-butyl­isophthalato)di-μ_3_-hydroxido-penta­zinc(II)]

**DOI:** 10.1107/S1600536809031365

**Published:** 2009-08-15

**Authors:** Dong-Sheng Zhou, Di Sun, Shi-Yao Yang, Rong-Bin Huang

**Affiliations:** a Department of Chemistry, Xiamen University, Xiamen 361005, People’s Republic of China

## Abstract

The asymmetric unit of the title compound, [Zn_5_(C_12_H_12_O_4_)_4_(OH)_2_(C_10_H_8_N_2_)]_*n*_, consists of three Zn^II^ ions (one of which is located on a twofold rotation axis), two 5-*tert*-butyl­isophthalate ligands, one 4,4′-bipyridine ligand and one hydroxide group. The five Zn^II^ ions form a penta­nuclear zinc cluster, which is further bridged by ten organic ligands, forming two-dimensional sheets. The central zinc ion of the cluster has site symmetry 2 and is octahedrally coordinated in a N_2_O_4_ donor set, whereas the other four zinc atoms are tetrahedrally coordinated by four O atoms. The coordination modes for the 5-*tert*-butyl­isophthalates are bis­(bidentate) or bidentate-monodentate. Hydrogen bonds are formed between adjacent sheets through the hydroxide groups and the O atoms of the monodentate carboxyl­ate groups.  The two *tert*-butyl groups are disordered over two positions with ratios of 0.64 (2):0.36 (2) and 0.85 (3):0.15 (3).

## Related literature

For general background to the structures and potential applications of isophthalic acid and its derivatives, see Li & Huang (2008 ▶); Ma *et al.* (2007 ▶); Pan *et al.* (2006 ▶); Yang *et al.* (2002 ▶, 2005 ▶). For related structures, see Li *et al.* (2004 ▶); Wang *et al.* (2005 ▶).
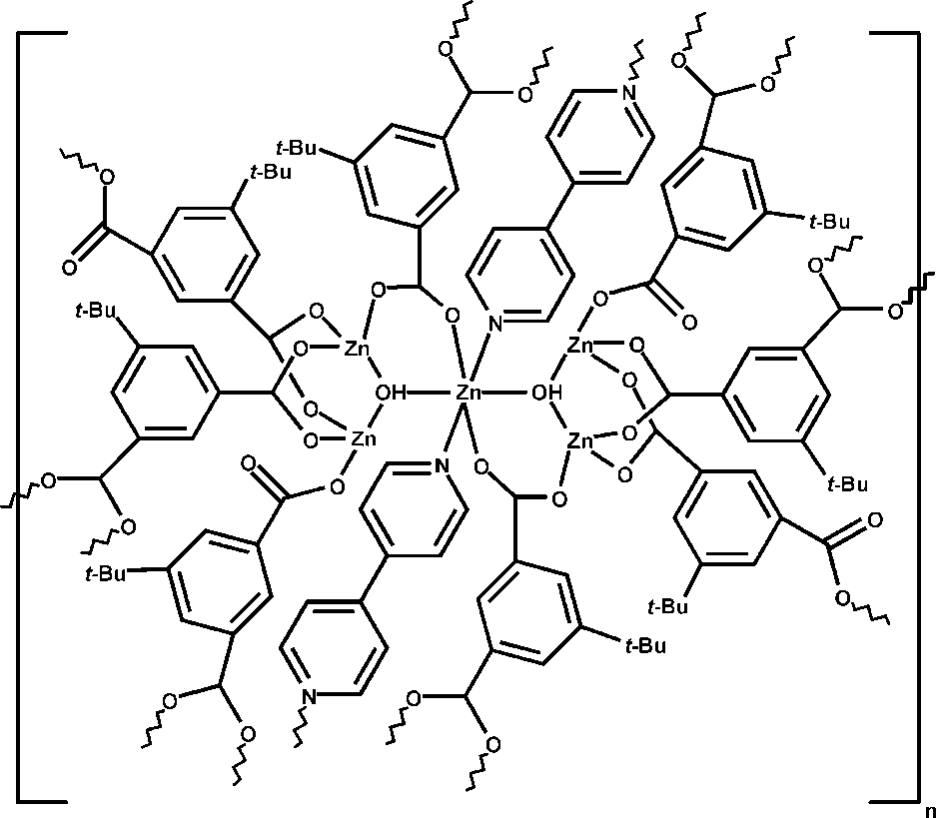


## Experimental

### 

#### Crystal data

[Zn_5_(C_12_H_12_O_4_)_4_(OH)_2_(C_10_H_8_N_2_)]
*M* *_r_* = 1397.91
Monoclinic, 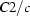

*a* = 26.1995 (5) Å
*b* = 11.2592 (2) Å
*c* = 19.6223 (4) Å
β = 102.0444 (18)°
*V* = 5660.87 (19) Å^3^
*Z* = 4
Mo *K*α radiation
μ = 2.16 mm^−1^
*T* = 173 K
0.34 × 0.30 × 0.20 mm

#### Data collection

Oxford Diffraction Gemini S Ultra diffractometer
Absorption correction: multi-scan (*CrysAlis RED*; Oxford Diffraction, 2007 ▶) *T*_min_ = 0.527, *T*_max_ = 0.671
13453 measured reflections
5494 independent reflections
4246 reflections with *I* > 2σ(*I*)
*R*_int_ = 0.031

#### Refinement

*R*[*F* ^2^ > 2σ(*F* ^2^)] = 0.028
*wR*(*F* ^2^) = 0.066
*S* = 1.00
5494 reflections
401 parameters
13 restraints
H atoms treated by a mixture of independent and constrained refinement
Δρ_max_ = 1.25 e Å^−3^
Δρ_min_ = −0.38 e Å^−3^

### 

Data collection: *CrysAlis CCD* (Oxford Diffraction, 2007 ▶); cell refinement: *CrysAlis RED* (Oxford Diffraction, 2007 ▶); data reduction: *CrysAlis RED*; program(s) used to solve structure: *SHELXS97* (Sheldrick, 2008 ▶); program(s) used to refine structure: *SHELXL97* (Sheldrick, 2008 ▶); molecular graphics: *ORTEPIII* (Burnett & Johnson, 1996 ▶); software used to prepare material for publication: *SHELXL97*.

## Supplementary Material

Crystal structure: contains datablocks I, global. DOI: 10.1107/S1600536809031365/dn2465sup1.cif

Structure factors: contains datablocks I. DOI: 10.1107/S1600536809031365/dn2465Isup2.hkl

Additional supplementary materials:  crystallographic information; 3D view; checkCIF report

## Figures and Tables

**Table 1:** Hydrogen-bond geometry (Å, °)

*D*—H⋯*A*	*D*—H	H⋯*A*	*D*⋯*A*	*D*—H⋯*A*
O9—H9⋯O7^i^	0.838 (10)	2.04 (2)	2.783 (3)	148 (3)

Symmetry code: (i) 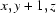.

**Fig. 1.**
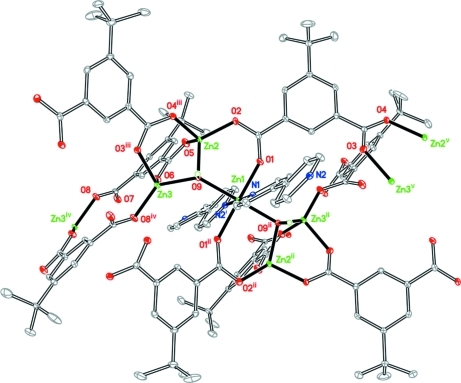
View showing the coordination environments of zinc ions in 1 with the atom labeling scheme. Ellipsoids are drawn at the 30% probability level. Hydrogen atoms on carbon atoms have been omitted for clarity. Symmetry codes: (i) x, y - 1, z; (ii) -x, y, -z + 1/2; (iii) x, -y + 1, z -1/2; (iv) -x, -y, -z; (v) x, -y + 1, z + 1/2.

**Fig. 2.**
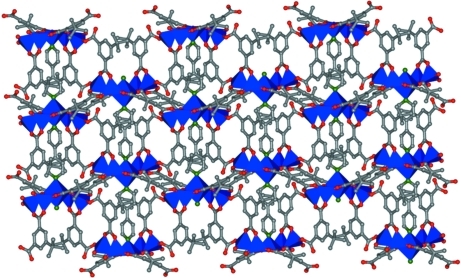
A perspective view of the two-dimensional sheets of 1 along a axis. Hydrogen atoms have been omitted for clarity.

**Fig. 3.**
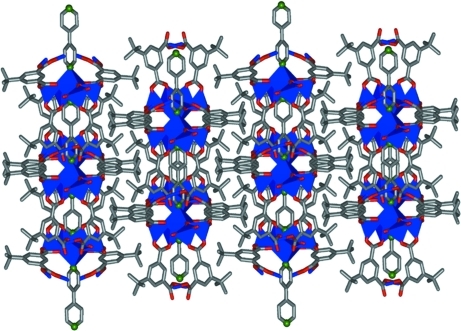
A perspective view of 1 along c axis. Hydrogen atoms have been omitted for clarity.

## supplementary crystallographic information

### Comment

Isophthalic acid and its derivatives have been used to construct coordination polymers. Some of these compounds display interesting structures and potential application properties (Li *et al.*, 2008; Ma *et al.*, 2007; Pan *et al.*, 2006; Yang *et al.*, 2002; Yang *et al.*, 2005). In this paper we report a coordination polymer [Zn_5_(µ_3_-OH)_2_(tbip)_4_(bpy)]_n_, 1 (tbip = 5-*tert*-butylisophthalate, bpy = 4,4'-bipyridine) synthesized by hydrothermal reaction.

The structure of 1 contains pentanuclear zinc clusters, (Fig. 1) in which each µ_3_-OH links three crystallographically unique Zn^II^ ions. The Zn^II^ ions exhibit two different coordination geometries: Zn1 coordinates to two µ_3_-OH moieties and two carboxylate oxygen atoms from two different tbips in the plane and two nitrogen atoms from two bpy ligands at the apexes giving a slightly distorted octahedral geometry; Zn2 or Zn3 atom is coordinated by three oxygen atoms from three tbips and one µ_3_-OH atom to complete a distorted tetrahedral environment. Coordination polymers with similar but different pentanuclear zinc clusters have been recently reported (Li *et al.*, 2004; Wang *et al.*, 2005). Two coordination modes for the tbips have been found: one is bis(bidentate), and the other one adopts bidentate and monodentate for each of its carboxyl groups. As a result, each pentanuclear zinc cluster is surrounded by ten organic ligands, eight tbips and two bpys. Each pentanuclear zinc cluster is further linked to six nearest-neighbors, forming a two-dimensional sheet parallel to *bc* plane (Fig. 2). The two-dimensional sheets are further packed along *a* axis (Fig. 3). Hydrogen bonds are formed between adjacent sheets by the hydroxyl groups and the oxygen atoms of the monodentate carboxyl groups.

### Experimental

The suspension of 5-*tert*-butylisophthalic acid (H_2_tbip, 0.045 g, 0.20 mmol) and 4,4'-bipyridine (bpy, 0.039 g, 0.20 mmol) in H_2_O (10 ml), and 25% tetramethylammonium hydroxide aqueous solution was slowly added until the pH of the solution was adjusted to 7, then Zn(NO_3_)_2_^.^6H_2_O (0.12 g, 0.40 mmol) was added. The mixture was placed in a 20 ml Teflon-lined vessel, heated to 170°C at the rate of 0.2°C/min, and kept at 170°C for 3 days, then slowly cooled down to room temperature at the rate of 0.1°C /min. Colorless platelet crystals (0.045 g, yield 64%) were separated by filtration, washed with deionized water and dried in air. Elemental Analysis: C_58_H_58_N_2_O_18_Zn_5_, found (calc.) C 49.82 (49.83), H 4.27 (4.18), N 1.99 (2.00). FTIR (KBr, cm^-1^): 3412(*s*), 2960 (*s*), 1610(*s*), 1552(*m*), 1369 (*m*), 1069 (w), 808 (w), 719 (w).

### Refinement

The position and *U*_eq_ of the hydroxyl H atom were refined with O—H distance restrained to 0.85 Å. The aromatic H atoms were generated geometrically (C—H 0.95 Å) and were allowed to ride on their parent atoms in the riding model approximations, with their temperature factors set to 1.2 times those of the parent atoms. The two *tert*-butyls were treated with disordered models, the C—C distances are restrained to 1.54 Å and the temperature factors of the methyl carbon atoms were set to be equal. The methyl H atoms were generated geometrically (C—H 0.98 Å) and were allowed to ride on their parent atoms in the riding model approximations, with their temperature factors set to 1.5 times those of the parent atoms.

### Crystal data

**Table:**

[Zn_5_(C_12_H_12_O_4_)_4_(OH)_2_(C_10_H_8_N_2_)]	*F*(000) = 2856
*M**_r_* = 1397.91	*D*_x_ = 1.640 Mg m^−^^3^
Monoclinic, *C*2/*c*	Mo *K*α radiation, λ = 0.71073 Å
Hall symbol: -C 2yc	Cell parameters from 7045 reflections
*a* = 26.1995 (5) Å	θ = 2.3–29.1°
*b* = 11.2592 (2) Å	µ = 2.16 mm^−^^1^
*c* = 19.6223 (4) Å	*T* = 173 K
β = 102.0444 (18)°	Block, colorless
*V* = 5660.87 (19) Å^3^	0.34 × 0.30 × 0.20 mm
*Z* = 4	

### Data collection

**Table:**

Oxford Diffraction Gemini S Ultra diffractometer	5494 independent reflections
Radiation source: fine-focus sealed tube	4246 reflections with *I* > 2σ(*I*)
graphite	*R*_int_ = 0.031
Detector resolution: 16.1903 pixels mm^-1^	θ_max_ = 26.0°, θ_min_ = 2.3°
CrysAlis RED, Oxford Diffraction scans	*h* = −32→32
Absorption correction: multi-scan (*CrysAlis RED*; Oxford Diffraction, 2007)	*k* = −13→13
*T*_min_ = 0.527, *T*_max_ = 0.671	*l* = −24→24
13453 measured reflections	

### Refinement

**Table:**

Refinement on *F*^2^	Primary atom site location: structure-invariant direct methods
Least-squares matrix: full	Secondary atom site location: difference Fourier map
*R*[*F*^2^ > 2σ(*F*^2^)] = 0.028	Hydrogen site location: inferred from neighbouring sites
*wR*(*F*^2^) = 0.066	H atoms treated by a mixture of independent and constrained refinement
*S* = 1.00	*w* = 1/[σ^2^(*F*_o_^2^) + (0.0319*P*)^2^] where *P* = (*F*_o_^2^ + 2*F*_c_^2^)/3
5494 reflections	(Δ/σ)_max_ = 0.001
401 parameters	Δρ_max_ = 1.25 e Å^−^^3^
13 restraints	Δρ_min_ = −0.38 e Å^−^^3^

### Special details

**Table:**

Experimental. (CrysAlis RED; Oxford Diffraction Ltd., Version 1.171.31.8 Empirical absorption correction using spherical harmonics, implemented in SCALE3 ABSPACK scaling algorithm.
Geometry. All e.s.d.'s (except the e.s.d. in the dihedral angle between two l.s. planes) are estimated using the full covariance matrix. The cell e.s.d.'s are taken into account individually in the estimation of e.s.d.'s in distances, angles and torsion angles; correlations between e.s.d.'s in cell parameters are only used when they are defined by crystal symmetry. An approximate (isotropic) treatment of cell e.s.d.'s is used for estimating e.s.d.'s involving l.s. planes.
Refinement. Refinement of *F*^2^ against ALL reflections. The weighted *R*-factor *wR* and goodness of fit *S* are based on *F*^2^, conventional *R*-factors *R* are based on *F*, with *F* set to zero for negative *F*^2^. The threshold expression of *F*^2^ > σ(*F*^2^) is used only for calculating *R*-factors(gt) *etc*. and is not relevant to the choice of reflections for refinement. *R*-factors based on *F*^2^ are statistically about twice as large as those based on *F*, and *R*- factors based on ALL data will be even larger.

### Fractional atomic coordinates and isotropic or equivalent isotropic displacement parameters (Å^2^)

**Table:**

	*x*	*y*	*z*	*U*_iso_*/*U*_eq_	Occ. (<1)
Zn1	0.0000	0.39344 (4)	0.2500	0.01133 (10)	
Zn2	0.112667 (11)	0.33542 (3)	0.193919 (15)	0.01162 (8)	
Zn3	0.027868 (11)	0.29870 (3)	0.072954 (15)	0.01269 (8)	
O1	0.07251 (7)	0.39814 (15)	0.32346 (9)	0.0160 (4)	
O2	0.14721 (7)	0.37581 (17)	0.28684 (9)	0.0204 (4)	
O3	0.08674 (7)	0.64886 (16)	0.53303 (9)	0.0179 (4)	
O4	0.14678 (7)	0.58893 (16)	0.62566 (9)	0.0175 (4)	
O5	0.11765 (7)	0.16380 (15)	0.18948 (10)	0.0209 (5)	
O6	0.04856 (7)	0.13238 (15)	0.10268 (9)	0.0190 (4)	
O7	0.03696 (7)	−0.41560 (16)	0.07271 (9)	0.0212 (5)	
O8	0.03202 (7)	−0.26648 (16)	−0.00443 (9)	0.0191 (4)	
O9	0.03909 (6)	0.39068 (16)	0.16139 (9)	0.0117 (4)	
H9	0.0417 (14)	0.4619 (13)	0.1502 (18)	0.053 (12)*	
N1	0.0000	0.5789 (3)	0.2500	0.0137 (7)	
N2	0.0000	1.2080 (3)	0.2500	0.0133 (7)	
C1	0.15186 (10)	0.4375 (2)	0.40284 (13)	0.0146 (6)	
C2	0.12650 (10)	0.4891 (2)	0.45008 (13)	0.0143 (6)	
H2A	0.0896	0.4966	0.4394	0.017*	
C3	0.15512 (10)	0.5302 (2)	0.51342 (13)	0.0153 (6)	
C4	0.20896 (10)	0.5139 (2)	0.52952 (14)	0.0190 (6)	
H4A	0.2281	0.5394	0.5736	0.023*	
C5	0.23537 (10)	0.4616 (2)	0.48317 (14)	0.0192 (6)	
C6	0.20612 (10)	0.4258 (2)	0.41891 (13)	0.0179 (6)	
H6A	0.2234	0.3928	0.3853	0.022*	
C7	0.12122 (10)	0.3999 (2)	0.33291 (13)	0.0138 (6)	
C8	0.12757 (10)	0.5938 (2)	0.56094 (13)	0.0146 (6)	
C9	0.29516 (11)	0.4496 (3)	0.50082 (15)	0.0298 (8)	
C13	0.09777 (10)	−0.0335 (2)	0.15251 (13)	0.0143 (6)	
C14	0.06872 (10)	−0.1106 (2)	0.10376 (13)	0.0151 (6)	
H14A	0.0425	−0.0802	0.0671	0.018*	
C15	0.07813 (10)	−0.2310 (2)	0.10888 (13)	0.0138 (6)	
C16	0.11761 (10)	−0.2758 (2)	0.16168 (13)	0.0142 (6)	
H16A	0.1234	−0.3590	0.1652	0.017*	
C17	0.14871 (10)	−0.2000 (2)	0.20925 (13)	0.0146 (6)	
C18	0.13744 (10)	−0.0788 (2)	0.20429 (13)	0.0164 (6)	
H18A	0.1574	−0.0259	0.2371	0.020*	
C19	0.08700 (10)	0.0960 (2)	0.14760 (13)	0.0142 (6)	
C20	0.04689 (10)	−0.3146 (2)	0.05614 (13)	0.0148 (6)	
C21	0.19303 (10)	−0.2492 (2)	0.26583 (14)	0.0187 (6)	
C25	0.02271 (10)	0.6417 (2)	0.30652 (14)	0.0175 (6)	
H25A	0.0392	0.5995	0.3471	0.021*	
C26	0.02324 (10)	0.7631 (2)	0.30859 (14)	0.0183 (6)	
H26A	0.0395	0.8032	0.3501	0.022*	
C27	0.0000	0.8278 (3)	0.2500	0.0154 (8)	
C28	0.0000	0.9584 (3)	0.2500	0.0139 (8)	
C29	0.03577 (10)	1.0240 (2)	0.29771 (14)	0.0212 (7)	
H29A	0.0615	0.9842	0.3313	0.025*	
C30	0.03423 (10)	1.1455 (2)	0.29664 (14)	0.0200 (6)	
H30A	0.0587	1.1876	0.3307	0.024*	
C10	0.3204 (3)	0.5629 (6)	0.4881 (6)	0.0346 (8)	0.64 (2)
H10A	0.3078	0.6268	0.5143	0.052*	0.64 (2)
H10B	0.3118	0.5815	0.4383	0.052*	0.64 (2)
H10C	0.3583	0.5553	0.5035	0.052*	0.64 (2)
C11	0.3152 (4)	0.3482 (11)	0.4639 (7)	0.0346 (8)	0.64 (2)
H11A	0.3003	0.2735	0.4765	0.052*	0.64 (2)
H11B	0.3533	0.3449	0.4778	0.052*	0.64 (2)
H11C	0.3050	0.3602	0.4135	0.052*	0.64 (2)
C12	0.3125 (3)	0.4137 (9)	0.5797 (4)	0.0346 (8)	0.64 (2)
H12A	0.2922	0.3448	0.5892	0.052*	0.64 (2)
H12B	0.3064	0.4804	0.6091	0.052*	0.64 (2)
H12C	0.3497	0.3935	0.5900	0.052*	0.64 (2)
C22	0.2377 (5)	−0.1604 (13)	0.2822 (5)	0.0257 (16)	0.85 (3)
H22A	0.2260	−0.0887	0.3027	0.039*	0.85 (3)
H22B	0.2490	−0.1394	0.2392	0.039*	0.85 (3)
H22C	0.2670	−0.1958	0.3153	0.039*	0.85 (3)
C23	0.1707 (3)	−0.2688 (9)	0.3319 (3)	0.0387 (18)	0.85 (3)
H23A	0.1565	−0.1939	0.3453	0.058*	0.85 (3)
H23B	0.1985	−0.2966	0.3699	0.058*	0.85 (3)
H23C	0.1429	−0.3284	0.3222	0.058*	0.85 (3)
C24	0.2143 (3)	−0.3666 (6)	0.2451 (5)	0.039 (2)	0.85 (3)
H24A	0.1865	−0.4264	0.2379	0.058*	0.85 (3)
H24B	0.2432	−0.3930	0.2821	0.058*	0.85 (3)
H24C	0.2268	−0.3559	0.2018	0.058*	0.85 (3)
C10'	0.3139 (5)	0.5649 (11)	0.4642 (9)	0.0346 (8)	0.36 (2)
H10D	0.3521	0.5689	0.4749	0.052*	0.36 (2)
H10E	0.2997	0.6365	0.4817	0.052*	0.36 (2)
H10F	0.3013	0.5596	0.4136	0.052*	0.36 (2)
C11'	0.3096 (7)	0.341 (2)	0.4596 (13)	0.0346 (8)	0.36 (2)
H11D	0.2862	0.2743	0.4637	0.052*	0.36 (2)
H11E	0.3457	0.3171	0.4787	0.052*	0.36 (2)
H11F	0.3059	0.3619	0.4104	0.052*	0.36 (2)
C12'	0.3191 (5)	0.4493 (16)	0.5762 (6)	0.0346 (8)	0.36 (2)
H12D	0.3041	0.3847	0.5992	0.052*	0.36 (2)
H12E	0.3122	0.5254	0.5968	0.052*	0.36 (2)
H12F	0.3569	0.4376	0.5826	0.052*	0.36 (2)
C22'	0.238 (3)	−0.164 (8)	0.296 (3)	0.0257 (16)	0.15 (3)
H22D	0.2245	−0.0978	0.3195	0.039*	0.15 (3)
H22E	0.2533	−0.1330	0.2580	0.039*	0.15 (3)
H22F	0.2647	−0.2066	0.3294	0.039*	0.15 (3)
C23'	0.173 (2)	−0.307 (5)	0.326 (2)	0.0387 (18)	0.15 (3)
H23D	0.1353	−0.2925	0.3199	0.058*	0.15 (3)
H23E	0.1909	−0.2715	0.3704	0.058*	0.15 (3)
H23F	0.1796	−0.3922	0.3267	0.058*	0.15 (3)
C24'	0.2222 (15)	−0.338 (4)	0.226 (2)	0.039 (2)	0.15 (3)
H24D	0.2303	−0.2990	0.1850	0.058*	0.15 (3)
H24E	0.1999	−0.4073	0.2113	0.058*	0.15 (3)
H24F	0.2546	−0.3641	0.2570	0.058*	0.15 (3)

### Atomic displacement parameters (Å^2^)

**Table:**

	*U*^11^	*U*^22^	*U*^33^	*U*^12^	*U*^13^	*U*^23^
Zn1	0.0133 (2)	0.0072 (2)	0.0128 (2)	0.000	0.00121 (17)	0.000
Zn2	0.01415 (15)	0.01061 (16)	0.00943 (15)	−0.00041 (12)	0.00094 (12)	−0.00046 (12)
Zn3	0.01343 (15)	0.01206 (17)	0.01123 (15)	−0.00074 (12)	−0.00054 (12)	−0.00065 (12)
O1	0.0153 (10)	0.0158 (10)	0.0149 (9)	−0.0012 (8)	−0.0016 (8)	−0.0011 (8)
O2	0.0185 (10)	0.0297 (12)	0.0123 (9)	−0.0010 (9)	0.0016 (8)	−0.0072 (8)
O3	0.0161 (10)	0.0217 (11)	0.0154 (9)	0.0052 (8)	0.0027 (8)	0.0017 (8)
O4	0.0153 (9)	0.0236 (11)	0.0126 (9)	0.0032 (8)	0.0008 (8)	−0.0054 (8)
O5	0.0270 (11)	0.0097 (10)	0.0218 (10)	0.0020 (8)	−0.0043 (9)	−0.0026 (8)
O6	0.0199 (10)	0.0126 (10)	0.0211 (10)	0.0022 (8)	−0.0037 (8)	0.0017 (8)
O7	0.0282 (11)	0.0104 (10)	0.0216 (10)	−0.0023 (8)	−0.0029 (9)	0.0005 (8)
O8	0.0192 (10)	0.0189 (11)	0.0156 (9)	−0.0038 (8)	−0.0048 (8)	0.0006 (8)
O9	0.0143 (9)	0.0083 (10)	0.0123 (9)	0.0004 (8)	0.0023 (7)	−0.0015 (8)
N1	0.0169 (16)	0.0092 (16)	0.0153 (16)	0.000	0.0042 (13)	0.000
N2	0.0146 (15)	0.0115 (16)	0.0145 (15)	0.000	0.0047 (13)	0.000
C1	0.0157 (13)	0.0130 (14)	0.0138 (13)	−0.0014 (11)	−0.0001 (11)	0.0002 (11)
C2	0.0138 (13)	0.0139 (14)	0.0146 (13)	0.0017 (11)	0.0015 (11)	0.0010 (11)
C3	0.0170 (14)	0.0157 (15)	0.0134 (13)	0.0002 (11)	0.0034 (11)	0.0011 (11)
C4	0.0183 (14)	0.0246 (16)	0.0119 (13)	−0.0028 (12)	−0.0018 (11)	−0.0047 (12)
C5	0.0120 (13)	0.0268 (17)	0.0183 (14)	−0.0006 (12)	0.0024 (11)	−0.0057 (12)
C6	0.0187 (14)	0.0213 (16)	0.0151 (14)	0.0001 (12)	0.0063 (11)	−0.0042 (12)
C7	0.0208 (14)	0.0071 (13)	0.0126 (13)	−0.0007 (11)	0.0018 (11)	0.0006 (11)
C8	0.0149 (13)	0.0135 (14)	0.0157 (13)	−0.0064 (11)	0.0035 (11)	−0.0020 (11)
C9	0.0153 (15)	0.048 (2)	0.0260 (17)	−0.0003 (14)	0.0031 (13)	−0.0138 (15)
C13	0.0174 (14)	0.0112 (14)	0.0145 (13)	0.0013 (11)	0.0036 (11)	0.0020 (11)
C14	0.0147 (13)	0.0149 (14)	0.0145 (13)	0.0018 (11)	0.0004 (11)	0.0033 (11)
C15	0.0139 (13)	0.0140 (15)	0.0134 (13)	−0.0012 (11)	0.0023 (11)	−0.0006 (11)
C16	0.0175 (13)	0.0094 (14)	0.0152 (13)	0.0014 (11)	0.0023 (11)	−0.0006 (11)
C17	0.0135 (13)	0.0132 (14)	0.0157 (13)	0.0000 (11)	0.0003 (11)	0.0010 (11)
C18	0.0162 (13)	0.0130 (14)	0.0177 (14)	−0.0015 (11)	−0.0015 (11)	−0.0028 (11)
C19	0.0172 (14)	0.0109 (14)	0.0153 (13)	0.0015 (11)	0.0051 (11)	0.0014 (11)
C20	0.0117 (13)	0.0162 (16)	0.0152 (13)	0.0007 (11)	−0.0002 (11)	0.0006 (12)
C21	0.0160 (14)	0.0148 (14)	0.0205 (14)	0.0009 (12)	−0.0072 (12)	0.0000 (12)
C25	0.0197 (14)	0.0133 (15)	0.0179 (14)	0.0001 (11)	0.0006 (12)	0.0012 (12)
C26	0.0207 (14)	0.0139 (15)	0.0196 (14)	−0.0019 (12)	0.0026 (12)	−0.0039 (12)
C27	0.0147 (19)	0.011 (2)	0.023 (2)	0.000	0.0079 (16)	0.000
C28	0.0143 (18)	0.011 (2)	0.0179 (19)	0.000	0.0076 (16)	0.000
C29	0.0216 (15)	0.0128 (15)	0.0254 (16)	0.0015 (12)	−0.0033 (13)	0.0031 (12)
C30	0.0200 (15)	0.0143 (15)	0.0222 (15)	−0.0014 (12)	−0.0034 (12)	−0.0008 (12)
C10	0.0152 (15)	0.0541 (19)	0.0319 (15)	−0.0008 (13)	−0.0013 (13)	−0.0144 (15)
C11	0.0152 (15)	0.0541 (19)	0.0319 (15)	−0.0008 (13)	−0.0013 (13)	−0.0144 (15)
C12	0.0152 (15)	0.0541 (19)	0.0319 (15)	−0.0008 (13)	−0.0013 (13)	−0.0144 (15)
C22	0.0210 (16)	0.0226 (18)	0.028 (4)	−0.0050 (14)	−0.008 (3)	0.004 (3)
C23	0.030 (2)	0.047 (5)	0.034 (2)	0.000 (4)	−0.0062 (17)	0.022 (3)
C24	0.036 (3)	0.020 (3)	0.047 (4)	0.013 (2)	−0.023 (2)	−0.006 (3)
C10'	0.0152 (15)	0.0541 (19)	0.0319 (15)	−0.0008 (13)	−0.0013 (13)	−0.0144 (15)
C11'	0.0152 (15)	0.0541 (19)	0.0319 (15)	−0.0008 (13)	−0.0013 (13)	−0.0144 (15)
C12'	0.0152 (15)	0.0541 (19)	0.0319 (15)	−0.0008 (13)	−0.0013 (13)	−0.0144 (15)
C22'	0.0210 (16)	0.0226 (18)	0.028 (4)	−0.0050 (14)	−0.008 (3)	0.004 (3)
C23'	0.030 (2)	0.047 (5)	0.034 (2)	0.000 (4)	−0.0062 (17)	0.022 (3)
C24'	0.036 (3)	0.020 (3)	0.047 (4)	0.013 (2)	−0.023 (2)	−0.006 (3)

### Geometric parameters (Å, °)

**Table:**

Zn1—N2^i^	2.088 (3)	C16—H16A	0.9500
Zn1—N1	2.088 (3)	C17—C18	1.395 (4)
Zn1—O1	2.1323 (16)	C17—C21	1.533 (3)
Zn1—O1^ii^	2.1323 (16)	C18—H18A	0.9500
Zn1—O9^ii^	2.1947 (18)	C21—C24	1.523 (5)
Zn1—O9	2.1947 (18)	C21—C22	1.523 (5)
Zn2—O2	1.9136 (17)	C21—C23'	1.54 (2)
Zn2—O5	1.9398 (18)	C21—C22'	1.54 (2)
Zn2—O4^iii^	1.9539 (18)	C21—C23	1.545 (6)
Zn2—O9	1.9992 (16)	C21—C24'	1.564 (19)
Zn2—Zn3	2.9223 (4)	C25—C26	1.368 (4)
Zn3—O8^iv^	1.8764 (17)	C25—H25A	0.9500
Zn3—O3^iii^	1.9609 (19)	C26—C27	1.390 (3)
Zn3—O9	1.9893 (17)	C26—H26A	0.9500
Zn3—O6	2.0022 (17)	C27—C26^ii^	1.390 (3)
O1—C7	1.251 (3)	C27—C28	1.470 (5)
O2—C7	1.270 (3)	C28—C29	1.391 (3)
O3—C8	1.258 (3)	C28—C29^ii^	1.391 (3)
O3—Zn3^v^	1.9609 (19)	C29—C30	1.369 (4)
O4—C8	1.266 (3)	C29—H29A	0.9500
O4—Zn2^v^	1.9539 (18)	C30—H30A	0.9500
O5—C19	1.276 (3)	C10—H10A	0.9800
O6—C19	1.260 (3)	C10—H10B	0.9800
O7—C20	1.226 (3)	C10—H10C	0.9800
O8—C20	1.290 (3)	C11—H11A	0.9800
O8—Zn3^iv^	1.8764 (17)	C11—H11B	0.9800
O9—H9	0.838 (10)	C11—H11C	0.9800
N1—C25	1.345 (3)	C12—H12A	0.9800
N1—C25^ii^	1.345 (3)	C12—H12B	0.9800
N2—C30^ii^	1.340 (3)	C12—H12C	0.9800
N2—C30	1.340 (3)	C22—H22A	0.9800
N2—Zn1^vi^	2.088 (3)	C22—H22B	0.9800
C1—C2	1.377 (4)	C22—H22C	0.9800
C1—C6	1.397 (4)	C23—H23A	0.9800
C1—C7	1.499 (3)	C23—H23B	0.9800
C2—C3	1.390 (3)	C23—H23C	0.9800
C2—H2A	0.9500	C24—H24A	0.9800
C3—C4	1.392 (4)	C24—H24B	0.9800
C3—C8	1.478 (4)	C24—H24C	0.9800
C4—C5	1.384 (4)	C10'—H10D	0.9800
C4—H4A	0.9500	C10'—H10E	0.9800
C5—C6	1.391 (3)	C10'—H10F	0.9800
C5—C9	1.538 (4)	C11'—H11D	0.9800
C6—H6A	0.9500	C11'—H11E	0.9800
C9—C10	1.482 (7)	C11'—H11F	0.9800
C9—C12'	1.482 (12)	C12'—H12D	0.9800
C9—C11	1.503 (10)	C12'—H12E	0.9800
C9—C11'	1.559 (16)	C12'—H12F	0.9800
C9—C12	1.573 (8)	C22'—H22D	0.9800
C9—C10'	1.611 (12)	C22'—H22E	0.9800
C13—C18	1.390 (3)	C22'—H22F	0.9800
C13—C14	1.394 (3)	C23'—H23D	0.9800
C13—C19	1.485 (3)	C23'—H23E	0.9800
C14—C15	1.377 (4)	C23'—H23F	0.9800
C14—H14A	0.9500	C24'—H24D	0.9800
C15—C16	1.396 (3)	C24'—H24E	0.9800
C15—C20	1.508 (3)	C24'—H24F	0.9800
C16—C17	1.395 (3)		
			
N2^i^—Zn1—N1	180.0	C17—C16—C15	121.0 (2)
N2^i^—Zn1—O1	91.42 (5)	C17—C16—H16A	119.5
N1—Zn1—O1	88.58 (5)	C15—C16—H16A	119.5
N2^i^—Zn1—O1^ii^	91.42 (5)	C16—C17—C18	117.8 (2)
N1—Zn1—O1^ii^	88.58 (5)	C16—C17—C21	120.8 (2)
O1—Zn1—O1^ii^	177.16 (10)	C18—C17—C21	121.4 (2)
N2^i^—Zn1—O9^ii^	89.19 (5)	C13—C18—C17	121.7 (2)
N1—Zn1—O9^ii^	90.81 (5)	C13—C18—H18A	119.2
O1—Zn1—O9^ii^	87.82 (6)	C17—C18—H18A	119.2
O1^ii^—Zn1—O9^ii^	92.22 (6)	O6—C19—O5	124.0 (2)
N2^i^—Zn1—O9	89.19 (5)	O6—C19—C13	118.5 (2)
N1—Zn1—O9	90.81 (5)	O5—C19—C13	117.4 (2)
O1—Zn1—O9	92.22 (6)	O7—C20—O8	126.4 (2)
O1^ii^—Zn1—O9	87.82 (6)	O7—C20—C15	120.8 (2)
O9^ii^—Zn1—O9	178.38 (10)	O8—C20—C15	112.8 (2)
O2—Zn2—O5	104.87 (8)	C24—C21—C22	108.6 (8)
O2—Zn2—O4^iii^	110.89 (8)	C24—C21—C17	112.6 (3)
O5—Zn2—O4^iii^	111.02 (8)	C22—C21—C17	110.4 (6)
O2—Zn2—O9	117.59 (8)	C24—C21—C23'	92.6 (19)
O5—Zn2—O9	111.42 (8)	C22—C21—C23'	119 (2)
O4^iii^—Zn2—O9	101.18 (7)	C17—C21—C23'	112 (2)
O2—Zn2—Zn3	159.36 (6)	C24—C21—C22'	111 (4)
O5—Zn2—Zn3	82.50 (5)	C17—C21—C22'	117 (3)
O4^iii^—Zn2—Zn3	83.33 (5)	C23'—C21—C22'	109 (3)
O9—Zn2—Zn3	42.76 (5)	C24—C21—C23	108.9 (3)
O8^iv^—Zn3—O3^iii^	112.44 (8)	C22—C21—C23	109.0 (5)
O8^iv^—Zn3—O9	132.46 (8)	C17—C21—C23	107.3 (3)
O3^iii^—Zn3—O9	101.90 (7)	C22'—C21—C23	99 (3)
O8^iv^—Zn3—O6	99.38 (7)	C22—C21—C24'	95.3 (18)
O3^iii^—Zn3—O6	102.14 (8)	C17—C21—C24'	104.2 (15)
O9—Zn3—O6	104.54 (7)	C23'—C21—C24'	113 (2)
O8^iv^—Zn3—Zn2	171.76 (6)	C22'—C21—C24'	100 (4)
O3^iii^—Zn3—Zn2	75.75 (5)	C23—C21—C24'	129.5 (17)
O9—Zn3—Zn2	43.02 (5)	N1—C25—C26	123.4 (2)
O6—Zn3—Zn2	77.40 (5)	N1—C25—H25A	118.3
C7—O1—Zn1	146.93 (18)	C26—C25—H25A	118.3
C7—O2—Zn2	120.80 (16)	C25—C26—C27	120.0 (3)
C8—O3—Zn3^v^	129.98 (17)	C25—C26—H26A	120.0
C8—O4—Zn2^v^	121.73 (16)	C27—C26—H26A	120.0
C19—O5—Zn2	125.86 (16)	C26^ii^—C27—C26	116.8 (3)
C19—O6—Zn3	129.49 (16)	C26^ii^—C27—C28	121.62 (17)
C20—O8—Zn3^iv^	128.72 (17)	C26—C27—C28	121.62 (17)
Zn3—O9—Zn2	94.23 (7)	C29—C28—C29^ii^	115.9 (3)
Zn3—O9—Zn1	133.63 (9)	C29—C28—C27	122.07 (17)
Zn2—O9—Zn1	109.25 (7)	C29^ii^—C28—C27	122.07 (17)
Zn3—O9—H9	106 (2)	C30—C29—C28	120.5 (3)
Zn2—O9—H9	105 (2)	C30—C29—H29A	119.8
Zn1—O9—H9	106 (3)	C28—C29—H29A	119.8
C25—N1—C25^ii^	116.6 (3)	N2—C30—C29	123.3 (2)
C25—N1—Zn1	121.72 (16)	N2—C30—H30A	118.4
C25^ii^—N1—Zn1	121.72 (16)	C29—C30—H30A	118.4
C30^ii^—N2—C30	116.6 (3)	C9—C10—H10A	109.5
C30^ii^—N2—Zn1^vi^	121.70 (16)	C9—C10—H10B	109.5
C30—N2—Zn1^vi^	121.70 (16)	C9—C10—H10C	109.5
C2—C1—C6	120.0 (2)	C9—C11—H11A	109.5
C2—C1—C7	119.6 (2)	C9—C11—H11B	109.5
C6—C1—C7	120.4 (2)	C9—C11—H11C	109.5
C1—C2—C3	119.8 (2)	C9—C12—H12A	109.5
C1—C2—H2A	120.1	C9—C12—H12B	109.5
C3—C2—H2A	120.1	C9—C12—H12C	109.5
C4—C3—C2	119.4 (2)	C21—C22—H22A	109.5
C4—C3—C8	121.7 (2)	C21—C22—H22B	109.5
C2—C3—C8	118.8 (2)	C21—C22—H22C	109.5
C5—C4—C3	121.9 (2)	C21—C23—H23A	109.5
C5—C4—H4A	119.0	C21—C23—H23B	109.5
C3—C4—H4A	119.0	C21—C23—H23C	109.5
C4—C5—C6	117.5 (2)	C21—C24—H24A	109.5
C4—C5—C9	121.0 (2)	C21—C24—H24B	109.5
C6—C5—C9	121.4 (3)	C21—C24—H24C	109.5
C5—C6—C1	121.3 (3)	C9—C10'—H10D	109.5
C5—C6—H6A	119.4	C9—C10'—H10E	109.5
C1—C6—H6A	119.4	H10D—C10'—H10E	109.5
O1—C7—O2	125.0 (2)	C9—C10'—H10F	109.5
O1—C7—C1	118.4 (2)	H10D—C10'—H10F	109.5
O2—C7—C1	116.6 (2)	H10E—C10'—H10F	109.5
O3—C8—O4	125.6 (2)	C9—C11'—H11D	109.5
O3—C8—C3	116.5 (2)	C9—C11'—H11E	109.5
O4—C8—C3	117.9 (2)	H11D—C11'—H11E	109.5
C10—C9—C12'	93.3 (5)	C9—C11'—H11F	109.5
C10—C9—C11	111.1 (7)	H11D—C11'—H11F	109.5
C12'—C9—C11	111.2 (8)	H11E—C11'—H11F	109.5
C10—C9—C5	110.8 (4)	C9—C12'—H12D	109.5
C12'—C9—C5	115.1 (6)	C9—C12'—H12E	109.5
C11—C9—C5	113.5 (4)	H12D—C12'—H12E	109.5
C10—C9—C11'	115.2 (11)	C9—C12'—H12F	109.5
C12'—C9—C11'	114.7 (11)	H12D—C12'—H12F	109.5
C5—C9—C11'	107.3 (7)	H12E—C12'—H12F	109.5
C10—C9—C12	109.8 (3)	C21—C22'—H22D	109.5
C11—C9—C12	102.8 (7)	C21—C22'—H22E	109.5
C5—C9—C12	108.4 (4)	H22D—C22'—H22E	109.5
C11'—C9—C12	104.9 (11)	C21—C22'—H22F	109.5
C12'—C9—C10'	110.0 (5)	H22D—C22'—H22F	109.5
C11—C9—C10'	103.2 (8)	H22E—C22'—H22F	109.5
C5—C9—C10'	102.7 (5)	C21—C23'—H23D	109.5
C11'—C9—C10'	105.9 (12)	C21—C23'—H23E	109.5
C12—C9—C10'	126.5 (5)	H23D—C23'—H23E	109.5
C18—C13—C14	119.4 (2)	C21—C23'—H23F	109.5
C18—C13—C19	120.7 (2)	H23D—C23'—H23F	109.5
C14—C13—C19	119.9 (2)	H23E—C23'—H23F	109.5
C15—C14—C13	119.9 (2)	C21—C24'—H24D	109.5
C15—C14—H14A	120.0	C21—C24'—H24E	109.5
C13—C14—H14A	120.0	H24D—C24'—H24E	109.5
C14—C15—C16	120.2 (2)	C21—C24'—H24F	109.5
C14—C15—C20	120.0 (2)	H24D—C24'—H24F	109.5
C16—C15—C20	119.7 (2)	H24E—C24'—H24F	109.5

Symmetry codes: (i) *x*, *y*−1, *z*; (ii) −*x*, *y*, −*z*+1/2; (iii) *x*, −*y*+1, *z*−1/2; (iv) −*x*, −*y*, −*z*; (v) *x*, −*y*+1, *z*+1/2; (vi) *x*, *y*+1, *z*.

### Hydrogen-bond geometry (Å, °)

**Table:**

*D*—H···*A*	*D*—H	H···*A*	*D*···*A*	*D*—H···*A*
O9—H9···O7^vi^	0.84 (1)	2.04 (2)	2.783 (3)	148 (3)

Symmetry codes: (vi) *x*, *y*+1, *z*.
